# Hospital for Sick Children, Great Ormond Street

**Published:** 1893-06-03

**Authors:** 


### FESTIVAL DINNERS AND MEETINGS.

### HOSPITAL FOR SICK CHILDREN, GREAT ORMOND STREET.

The forty-first annual general meeting of the Governors and subscribers of this hospital was held on Tuesday, May 30th, Viscount Gort (Vice-President) in the chair. Amongst the Governors present were: Mr. Arthur Lucas, Mr. John Murray, Dr. Sturges, Mr. G. L. Phipps Eyre, the Rev. F. A. Minnitt, Mr. Costell May, and Mr. Charles Barry. The report was unanimously adopted. In proposing a vote of thanks to the members of the medical staff for their very valuable services during the past year, Mr. Lucas alluded to the serious loss they had sustained in the death of Dr. Hadden, Assistant Physician to this hospital and to St. Thomas's. He was one of those who might truly be called a hero in doing his duty, and it was, he believed, through his devotion to his work that he had contracted his last illness. Mr. Lucas went on to make a proposal, which was cordially agreed to, that Baron Hirsch, in recognition of his generous gifts to the hospital, amounting to £2,200, should be elected a Vice-President. An earnest appeal was made for funds to enable the work of the hospital to be satisfactorily carried on. The new wing, which is now partly occupied, will be formally opened on June 24th, when it is to be hoped the friends of the hospital will come forward with substantial help, there being still a debt of £12,000 to defray. There had been a serious decrease in subscriptions and donations, and in consequence the committee had been compelled to apply most of the legacies they had received during the year as income and not as capital. Various members of the committee having been re-elected, and slight alterations made in some of the rules, the meeting closed with a cordial vote of thanks to the Chairman.

